# Experimental Models for Rare Melanoma Research—The Niche That Needs to Be Addressed

**DOI:** 10.3390/bioengineering10060673

**Published:** 2023-06-01

**Authors:** Ioana Ionita, Daniel Malita, Cristina Dehelean, Emilian Olteanu, Iasmina Marcovici, Andreea Geamantan, Sorin Chiriac, Andrea Roman, Daniela Radu

**Affiliations:** 1 Faculty of Medicine, “Victor Babes” University of Medicine and Pharmacy, Eftimie Murgu Square No. 2, 300041 Timisoara, Romania; 2 Faculty of Pharmacy, “Victor Babes” University of Medicine and Pharmacy, Eftimie Murgu Square No. 2, 300041 Timisoara, Romania; 3 Research Center for Pharmaco-Toxicological Evaluations, Faculty of Pharmacy, “Victor Babes” University of Medicine and Pharmacy, Eftimie Murgu Square No. 2, 300041 Timisoara, Romania; 4 Center for Research and Innovation in Personalized Medicine of Respiratory Diseases, “Victor Babes” University of Medicine and Pharmacy, Eftimie Murgu Square No. 2, 300041 Timisoara, Romania

**Keywords:** uveal melanoma, acral lentiginous melanoma, mucosal melanoma, in vitro, in vivo, in ovo, models, next-generation sequencing, CRISPR/Cas9

## Abstract

Melanoma, the tumor arising from the malignant transformation of pigment-producing cells—the melanocytes—represents one of the most severe cancer types. Despite their rarity compared to cutaneous melanoma, the extracutaneous subtypes such as uveal melanoma (UM), acral lentiginous melanoma (ALM), and mucosal melanoma (MM) stand out due to their increased aggressiveness and mortality rate, demanding continuous research to elucidate their specific pathological features and develop efficient therapies. Driven by the emerging progresses made in the preclinical modeling of melanoma, the current paper covers the most relevant in vitro, in vivo, and in ovo systems, providing a deeper understanding of these rare melanoma subtypes. However, the preclinical models for UM, ALM, and MM that were developed so far remain scarce, and none of them is able to completely simulate the complexity that is characteristic to these melanomas; thus, a continuous expansion of the existing library of experimental models is pivotal for driving advancements in this research field. An overview of the applicability of precision medicine in the management of rare melanoma subtypes is also provided.

## 1. Introduction

Melanoma is a complex pathology with various subtypes, being included in extensive research studies due to its aggressiveness and continuous progression [1]. Melanoma arises from the malignant transformation of melanocytes, which are pigment-producing cells present in the skin [2,3], eyes, ears, or digestive epithelium [4]. This complex pathology is not limited to cutaneous melanoma but also comprises several other extracutaneous subtypes, including uveal, acral, and mucosal melanomas [1,5].

Uveal melanoma (UM) is a collective term for melanomas involving the iris of the eye, ciliary body, and choroid [5]. Compared to CM, UM has a lower mutational burden, and some therapies that show success in CM treatment are relatively ineffective in UM [6]. Acral lentiginous melanoma (ALM) also belongs to the melanoma group as a subtype with a relatively low incidence (about 2–3% of all cases), being developed on palms, soles, and nails [7]. Unlike CM, the appearance of ALM is not related to exposure to ultraviolet (UV) radiation, because its development is found in sun-protected zones [8]. Another aggressive subtype of melanoma is mucosal melanoma (MM), which originates from the melanocytes of the tissues lining the respiratory, gastrointestinal, or urogenital tracts [9]. MM differs from CM in terms of molecular and clinical features, presenting a higher genomic instability [10].

Experimental models remain useful tools in cancer research not only by providing a broad understanding of disease progression, invasion, molecular basis, tumor heterogeneity, and microenvironment, but also by facilitating the development of efficient therapies [11]. Cancer research routinely includes the use of in vitro, in vivo, and in ovo models [12,13,14]. Based on their complexity, in vitro models comprise a variety of experimental systems, from conventional 2D models based on monolayer cell cultures to more advanced 3D models (e.g., spheroids, organoids, organ-on-a-chip, etc.) that mimic the tumor microenvironment and provide a much closer similarity to the in vivo physiological conditions [15,16]. In vitro models are used for gathering relevant information on various processes such as cell proliferation, invasion, signaling pathways, and tumor sensitivity or resistance to treatment [15,17]. In vivo models, which are based on the experimentation on living animals (e.g., rodents, zebrafish, etc.) are valuable research tools providing substantial insights into tumor biology, etiology, disease pathology, genetic mutations, and therapeutic options [15]. The in ovo models, in particular the chick embryo chorioallantoic membrane (CAM), also serve as relevant systems for preclinical oncological research, allowing the investigation of cancer progression and treatment efficacy [14].

Throughout this paper, we will discuss the detailed histopathology of rare melanomas and their models used for research, trying to compare the different models based on their approximation to rare melanomas in humans and the information that can be obtained using the models in question. Finally, precision medicine is portrayed as a state-of-the-art treatment approach for future rare melanoma management.

## 2. Histopathological Description of Rare Melanoma Subtypes

### 2.1. General Aspects

Histologically, melanoma is characterized by a proliferation of atypical melanocytes that exhibit a range of morphological features. These features include an enlarged pleomorphic nucleus, a prominent nucleolus, and abundant cytoplasm that may contain melanin pigment. Melanoma cells are typically arranged in nests or cords within the dermis or subcutaneous tissue, although they may also exhibit a diffuse growth pattern. The cells may be surrounded by a fibrous stroma and may invade surrounding tissues. One important feature of melanoma is the presence of melanin pigment, which can be observed on histological examination. Melanin pigment may be seen within individual melanoma cells or within tumor-infiltrating macrophages, which are phagocytose melanoma cells that have undergone necrosis. In addition to the presence of atypical melanocytes, melanoma may also exhibit a range of other histological features that can aid in diagnosis and classification. These features include the presence of mitotic figures, the degree of tumor invasion, the presence of ulceration, and the presence of lymphovascular invasion [2,3]. 

### 2.2. Characteristics of UM

UM is the most common type of primary intraocular cancer in adults, often caused by GNAQ or GNA11 mutations instead of the BRAF or NRAS mutations found in cutaneous melanomas (CM). Individuals with fair skin, light-colored eyes, congenital ocular melanocytosis, ocular melanocytoma, or the BAP1-tumor predisposition syndrome are at higher risk. Treatment for UM aims to preserve the eye and useful vision while preventing metastasis, and enucleation is now less common than radiotherapy, phototherapy, or local tumor resection. However, around 50% of patients eventually develop metastatic disease, which typically affects the liver and is often fatal within a year. Although UM metastases are less responsive to chemotherapy and immune checkpoint inhibitors than CM, new treatments such as partial hepatectomy, percutaneous hepatic perfusion with melphalan, or tebentafusp may offer hope, as the understanding of tumor immunology and metabolism continues to improve [18].

### 2.3. Characteristics of ALM

ALM is a type of melanoma that occurs on acral skin, such as palms, soles, and nail beds. It is a relatively uncommon subtype, but it is the most common type found in individuals with darker skin, such as those of Asian or African descent [19]. Because of the mild changes to the histopathological and clinical level, early diagnosis of this type of melanoma is quite difficult, but dermoscopy is the basic tool for detection [20]. Delayed diagnosis and advanced disease at presentation lead to a poor prognosis for ALM patients [19]. The cause of ALM is not well understood, but trauma or chronic inflammation are proposed as possible causes. ALM tends to present with fewer atypical nevi and a lower incidence of sunburn compared to superficial spreading melanoma (SSM) but has a higher incidence of personal and family history of non-cutaneous malignancies. The diagnosis of ALM, especially subungual lesions, can be difficult, and an adequate biopsy specimen is crucial. Histologically, ALM consists of confluent dendritic or epithelioid melanocytes found along the dermal-epidermal junction, with upward pagetoid migration and dermal invasion presenting as atypical epithelioid nests or cords. ALM has a lower incidence of activating mutations in BRAF and PTEN, but a higher incidence of activating mutations or amplifications of wild-type KIT, a type of receptor tyrosine kinase, compared to SSM or nodular CM [19]. 

### 2.4. Characteristics of MM

Primary MM are melanomas that arise from mucosal surfaces. They are rare, accounting for only 0.8–3.7% of all melanomas, and must be differentiated from metastases from ocular and CM. Predominantly MM are divided into site-specific of presumed primary, like MMs of the head and neck, MMs of the anorectal region, MMs of the vulva and vagina, primary urinary tract MMs, and MMs of the penis [21]. Comparative genomic hybridization analysis conducted on primary melanomas located on acral skin, mucosa, and skin with chronic sun-induced damage revealed that there were focal amplifications in chromosome 4q12.7. Among the receptor tyrosine kinases located at 4q12, KIT was hypothesized to be an oncogene. KIT encodes a type III transmembrane receptor tyrosine kinase that has activating mutations in either the intracellular juxtamembrane or kinase domains in a subset of melanomas. Studies have shown that 14% of MM harbor activating KIT mutations, while the prevalence of BRAF and NRAS oncogenic mutations in MM is much lower [21]. The prevalence of BRAF mutations is reported to be between 56% and 59% in CM, whereas it is only 5% for BRAF and 14% for NRAS oncogenic mutations in MM. Further, genetic factors that may be involved in the pathogenesis and progression of mucosal melanomas, are mutations in GNAQ/11. MM may be less immunogenic than CM, possibly due to a lower mutational burden [22]. Because of the areas where this subtype of melanoma develops, the diagnosis is more difficult and delayed. Therefore, spread and metastasis are frequently reported [8].

## 3. Current Status of Rare Melanoma Experimental Models

### 3.1. General Aspects

Before addressing the types of models used for rare melanoma research, first we need to have a look at the models currently available for the most common form of melanoma, which is CM. The primary, or better yet, the most-used models for melanoma research are in vivo models—genetically engineered models (GEMs), syngeneic transplantation models (STMs), patient-derived xenografts (PDXs), zebrafish melanoma models (ZMMs)—and their alternatives that are advanced but non-animal (or in vitro) models—cell cultures, skin reconstructs, tissues explants, and three-dimensional (3D) spheroids and organoids [23,24]. Most preliminary studies investigating melanoma biology or conducting drug screening are performed using in vitro 2D cultures. Furthermore, although monolayers do not recapitulate the complex cell–cell and cell–extracellular matrix (ECM) interplay, they can be successfully used to develop more advanced models—spheroids, human skin equivalents, melanoma-on-a-chip, etc.—that provide a high-fidelity imaging of the tumor microenvironment [23].

The use of GEMs in melanoma research has significantly improved our understanding of the functional genetics involved in melanoma formation and progression. These models are designed to carry one or more known drivers of specific subtypes of human melanoma, allowing for the study of how these genetic alterations contribute to the disease. GEM melanoma models have been found to have more physiological growth rates compared to other models. This makes them highly useful for evaluating therapies and sunscreens, especially small-molecule drugs such as BRAF V600E and MEK inhibitors that exhibit response characteristics like human melanoma. With the advent of immune-oncology therapies as standard melanoma treatment, there is potential for GEM melanoma models to be employed in the evaluation of these therapies as well. Therefore, numerous studies have been conducted to explore the use of GEM melanoma models in preclinical drug discovery and development [24,25]. STMs, mainly mouse models, are tumors derived from murine cancer cells that have been engrafted on genetically identical mouse strains. The main line of mice that represent the source of most derived melanoma cell lines is represented by C57BL/6 [26]. B16 melanoma in C57BL/6 mice is the most used STM for studying melanoma, as it has high metastatic potential and rapid growth. However, these models do not exhibit the chronic inflammatory environment present in human melanoma and may not represent the genetic diversity of human melanoma, as they originate from a single mouse strain. Despite these limitations, syngeneic models are useful for studying interactions between melanoma cells and immune cells and identifying immunotherapies targeting melanoma-associated antigens [27].

The use of PDXs, which can be viewed as a basic transplant model in which human melanoma cancer cells from patients are injected into immunocompromised mice, has opened up novel avenues regarding important aspects of melanogenesis and the appearance and function of drug resistance in melanoma. While PDXs have provided valuable data, they do present strong limitations when viewed in detail, such that the majority of samples used are derived from metastatic sites. PDXs, once injected into the mouse host, shift from their human tumor-stromal component and tumor-associated immune microenvironment to that of the host mouse, thus introducing a myriad of variables whose probability changes the outcome of the data [24,28].

ZMMs have been found to display similarities to human melanoma at the genomic level. Although the mutation frequencies in zebrafish tumors are different from those seen in sun-exposed human melanoma, zebrafish tumors do exhibit high levels of copy number variation, which is also a common feature in human melanoma [24]. Many genes subject to copy number variation in zebrafish melanoma are similarly varied in their copy number in human melanoma, suggesting that these alterations play an important role in the initiation and progression of both types of melanomas. The similarities between zebrafish and human melanomas in terms of pathway activity changes have made zebrafish melanomas a valuable platform for investigating genetic and environmental melanoma modifiers that contribute to initiation, progression, metastasis, and treatment [29].

One of the difficulties encountered during cancer research is choosing a proper experimental model that best reflects the tumor system that is being studied [11]. Thus, an important aspect that should be considered when selecting the most fitting model for rare melanoma research is that CM presents distinct features from non-cutaneous melanoma types which also differ significantly from each other in terms of behavior, clinical outcome, incidence, and genetic drivers [30,31]. To facilitate the model selection process, a comparative presentation of the main characteristics retained by CM, UM, ALM, and MM is shown in Table 1. 

**Figure 1:**
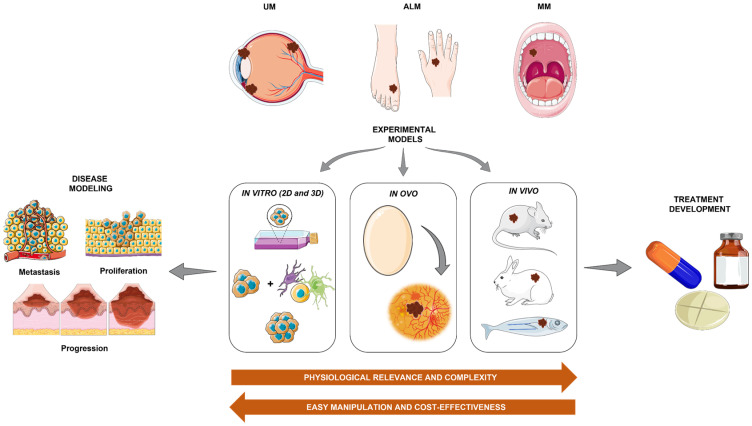
Schematic illustration of the main experimental models useful for disease modeling and treatment development in UM, ALM, and MM preclinical research. This figure was created using https://smart.servier.com/ (accessed on 4 April 2023). UM = uveal melanoma; ALM = acral lentiginous melanoma; MM = mucosal melanoma; 2D = two-dimensional; 3D = three-dimensional.

Further, the identified preclinical models used during the research of the three rare melanoma subtypes discussed throughout this review are illustrated in Figure 1, and separately detailed in Section 3.2, Section 3.3 and Section 3.4.

### 3.2. Experimental Models for UM

Efforts have been focused on investigating the underlying molecular and cellular processes that contribute to the development and metastasis of UM, to develop effective therapeutic approaches. To date, most in vitro studies exploring UM biology, development, progression, and metastasis, or searching for novel efficient treatments, are usually based on established UM cell lines [41,42], which remain essential tools during the preclinical investigation of disease pathogenesis and drug development [43]. To exemplify a few studies, Alvarez et al. demonstrated the anti-cancer effect of mifepristone (MF), using both primary (92.1, MP41, MP46, MEL270) and metastatic (OMM2.5) UM cell lines. Their results proved the efficiency of MF in all tested cell lines, an effect that was independent from cell line genetic burden, donor, or clinical history [44]. Another in vitro study performed on primary (92.1, MEL270), and metastatic (OMM1, OMM2.3, OMM2.5) UM cell lines showed that fucoidan exerted a cell type-specific effect by reducing the proliferation of MEL270 cells, increasing the proliferation of 92.1 and OMM2.5, and having no impact on the proliferation of OMM1 and OMM2.3 cells [45].

Despite the considerable availability of UM in vitro models, a crucial aspect that needs to be considered when choosing the right model for preclinical studies is that some UM cell lines present genetic variations leading to an improper representation of the original tumor. For instance, UM presents a distinct genetic background compared to CM [41]; however, some cell lines derived from primary (OCM-1, OCM-3, OCM-8, SP6.5) or metastatic (MUM2C) UM were discovered to lack GNAQ and GNA11 mutations (found in 80% of UMs), while carrying BRAF (V600E) mutations, which are specific to CM [18,43]. 

The emergence of 3D in vitro models contributed to the development of more advanced and physiologically relevant systems for cancer study [42], including UM. One factor that influences melanoma behavior in the tumor microenvironment (TME), especially its cellular components which might contribute to cancer progression and resistance to therapy [46]. Particular attention was given to the influence of TME on UM behavior using established 3D models. For example, Anfuso et al. developed a novel in vitro model by co-culturing 92.1 UM cells with human retinal pericytes (HRPC), to simulate the interaction between TME and cancer cells. The authors describe a bidirectional crosstalk between the co-cultured cells—UM cells induced morphological changes in HRPC, stimulated their proliferation and motility, and promoted their transition into cancer-activated fibroblasts (CAF) by elevating the production of diffusible factor PDGF-B, while HRPC increased the invasiveness of UM cells by upregulating the active MMP9 isoform. They also showed that imatinib effectively prevented the changes induced by 92.1 cells [35]. In another study, Babchia et al. showed that the co-culture of Mel270 and OMM2.3 UM cells with LX-2 activated hepatic stellate cells resulted in prominent transcriptional changes and an increase in the production of inflammatory mediators, without harming the proliferation of UM cells. Moreover, they discovered that metastatic UM cells were more responsive to LX-2 cells’ paracrine signaling compared to non-metastatic UM cells [47]. 

Tumor spheroids were also developed using established primary and metastatic UM cell lines (i.e., 92.1, Mel270, UPMD2, UPMD3, MP46, MM28, OMM1) as complex models for investigating a potential treatment option for UM based on electrochemotherapy [36,37]. The authors described the obtained spheroids as presenting different features, depending on the cell line: 92.1 spheroids presented an increasing size and compactness, Mel270 spheroids were large and flat, with low compactness and increasing size, while the UPMD-derived spheroids were small and presented constant size and compactness [37].

Another preclinical model system that has been extensively studied for this purpose is the chick embryo. The highly vascularized CAM of the chick embryo provides a tissue composition that allows easy visualization and tracking of grafted tumor cells, making it an attractive experimental model system. Two approaches have been developed using this model: (1) direct inoculation of tumor cells onto the CAM, resulting in rapid tumor formation and spontaneous metastasis, and (2) intravenous inoculation of tumor cells to study their ability to colonize internal organs. Validating this model system in UM would enhance research resources for studying the steps necessary for establishing metastases at distant sites in the body [48]. A previous paper presented the development of Matrigel grafts on chick embryo CAM using the 92.1 UM cell line—a suitable model for CAM assays due to its high pigmentation grade which makes the grafts observable without artificial cell labeling—for the investigation of UM tumor growth and electrochemotherapy as a potential treatment for this rare melanoma subtype [36]. 

Mice are the most cost-effective mammalian model due to their fecundity, gestation time, and size, and have been widely used in UM studies. Genetic manipulation of mice has produced various strains used in many UM models [34]. The STMs mouse model has been used extensively in UM research. In this model, melanoma cells from a cutaneous origin are implanted in mice that share the same genetic background as the mice from which the line was derived. Although not uveal in origin, this model mimics the behavior of UM in humans and allows for the investigation of the entire metastatic process. The ability to study the interaction between tumor and host cells as cancer progresses in an immunocompetent animal is the greatest strength of this model. The most commonly used STM involves inoculating C57BL/6 mice with the B16LS9 cell line, which metastasizes to the liver. However, a primary disadvantage of this model is that available mouse melanoma cell lines are of cutaneous origin, so their response to therapy and molecular drivers may differ from those observed in human UM. Nevertheless, there are a few STM models that carry canonical UM mutations, and if mouse UM cell lines could be derived from genetically engineered mouse models, they would be powerful tools for STM models [27,34]. 

While mouse models are commonly used in studying UM, other animal models also have their advantages. For example, rabbits have large eyes which make implantation and monitoring of tumor cells easy. Zebrafish models of UM are beneficial for high-throughput pharmacologic screening and in vivo microscopy, although assessing metastasis is difficult due to the induction of multiple primary tumors and the requirement of *p53* mutation [34]. Zebrafish was described as a relevant preclinical model for anti-UM drug screening and investigation of UM cells’ behavior. Recently, van der Ent et al. observed that UM cell lines exerted a differential phenotype following injection into zebrafish embryos: cells derived from primary tumors (Mel270) showed a lower metastatic and proliferation potential compared to those derived from metastases (OMM2.3, and OMM2.5). Mel270 also showed a lower tumor burden compared to another primary tumor cell line 92.1, and lower migratory potential compared to the metastatic OMM1 cell line [49]. Tobia et al. developed a UM orthotopic model by injecting 92.1 cells into the eye of zebrafish embryos which was applied for the assessment of the anti-tumor effect exerted by three conventional chemotherapeutic drugs (i.e., paclitaxel, panobinostat, and everolimus) [50].

The main preclinical models used in UM research are summarized in Table 2.

### 3.3. Experimental Models for ALM

Acral skin, found on the extremities, is distinct from the skin on other parts of the body due to several unique features. These include the lack of hair follicles and sebaceous glands, a thick stratum corneum, hypopigmentation, and encapsulated mechanoreceptors. In addition, acral skin has a unique grooved surface that alternates between ridges and sulci, with eccrine ducts located at the center of the ridges. The number and location of melanocytes vary in different areas of the body. Acral skin, such as the palms and soles, contains between 40 and 270 melanocytes per 1 mm length of the epidermal/dermal junction, while the nail apparatus has less than 20 melanocytes per 1 mm length. Melanocytes in acral skin are in the two lowest layers of the epidermis, while in non-acral skin, they are mainly found in hair follicles. Melanocyte precursors were also detected in the secretory portion of eccrine glands. Additionally, low-affinity nerve growth factor receptor (NGFR)-positive dermal stem cells isolated from glabrous human foreskin are able to differentiate into melanocytes. Differences in gene expression profiles, such as the enhanced expression of keratin 9, 6, and 16 in acral skin, suggest that the microenvironment may play a role in regulating melanogenesis. It is conceivable that melanomas arising in acral locations may be influenced by these microenvironment differences, as well as external environmental factors, such as UV radiation and trauma. Furthermore, the type of skin can impact the inflammatory response, which may also influence cancer development [53]. As such, unfortunately, models for ALM have not progressed as rapidly as those available for CM. STMs specific for ALM do not exist, but several PDXs platforms have been developed, including PDXs for ALM. Until recently, there have been only a few ALM cell lines available for study, but additional cell lines have been developed, most derived from PDXs. These cell lines have been at least partially genetically characterized, but the number of available cell lines is still limited [28,53]. The scarcity of in vitro models for ALM might be attributed to the rarity of ALM cases in the regions where melanoma is extensively studied, but also to the difficulties that are frequently encountered during cell line establishment, since ALM cells have a less aggressive growth in culture, and require specific growth factors to proliferate compared to CM cells [53]. Despite the reduced number of existing preclinical models for ALM, several cell lines—derived from the either primary lesions (e.g., SM3, WM3211, MMG1, and SMYM-PRGP), or metastases (e.g., SM2-1, and Mel18)—have been developed so far as in vitro models for this rare melanoma type. By providing complex profiling of some of the existing ALM cell lines (i.e., WM3211, MMG1, SMYM-PRGP, SM2-1, Mel18, Mel-2), Furney et al. determined their genetic similarities to ALM tumor samples, concluding that they are valuable models in ALM research [39].

Drug intervention experiments conducted with melanoma PDXs and derived cell lines have shown that these models can be used to identify the causal role of genetic alterations in therapy resistance and may be used to test drug combinations. Genetic alterations in TERT have been found in more than 40% of acral melanomas, and TERT inhibition has been shown to decrease cell viability in ALM cell lines with TERT mutations, making it a potential therapeutic target. KIT mutations have been found in ALM, and KIT inhibition has been shown to decrease the viability of WM3211 cells [28,53]. Hu et al. recently reported the establishment of four patient-derived primary ALM cell lines (XYAM-1, -2, -3, and -4) which tested positive for S100 and vimentin. Three cell lines (XYAM-1, -3, and -4) were found to harbor TP53 mutation, while only one presented KRAS and TERT mutations (HYAM-4). KIT mutation was found only in XYAM-1 cells. An ALM xenograft was also established in vivo by the subcutaneous injection of XYAM-4 cells in NSG mice for assessing their metastatic potential [40]. 

The experimental models used in ALM research are presented in Table 3.

### 3.4. Experimental Models for MM

There is a lack of models for MM. One reason for this is likely due to the fact that MM represents approximately 1% of all diagnosed cases of melanoma [55,56]. Nevertheless, MM prognosis is dismal, and it tends to present with aggressive clinical features, with a 5-year survival rate, considering all stages at the time of diagnosis, of 14%, which is considerably lower than the 80% survival rate for CM [56]. This highly aggressive biological behavior is likely exacerbated by the fact that the initial lesions are in low visibility areas and that the mucosa where these melanomas arise have different stroma than CM so that MM can be highly invasive at the time of diagnosis and already have metastasis.

However, it is worth mentioning that previous studies reported the successful establishment of patient-derived MM cell lines. For example, Lourenço et al. established a novel cell line (MEMO) derived from an oral MM which showed similar features to melanoma cells in terms of morphology (i.e., spindle, polyhedral, and star-like shaped), ultrastructure (i.e., presence of melanosomes and dark cytoplasmatic granules), and marker expression (i.e., S-100, HMB-45, and Melan-A). The cells also presented an increased replication in culture [57]. In another study, two cell lines (COMM-1 and COMM-2) presenting the Melan-A marker of melanoma and melanin granules were established. The cells presented two heterogeneous phenotypes, adherent (with a spindle-like morphology) and suspension cells (with a round shape, and a higher metastatic capacity), respectively. These cell lines were used to investigate the possible link between cancer heterogeneity and metastasis in melanoma [58]. Shi et al. established the MM9H-1 cell line from the tumor tissue derived from a patient with MM. The cells were spindle-shaped, presented a high expression of melanoma markers (i.e., S-100, HMB45, Melan-A, Tyrosinase), and produced melanin in high quantities. The morphology, proliferation, and marker expression were maintained during cell passaging. The cells were also able to rapidly generate spheroids during culture in ultra-low attachment plates. This cell line was also successfully used to establish a PDX in BALB/c nude immunodeficient mice which served as a model for the evaluation of the anti-cancer effect of some therapeutic agents (i.e., palbociclib, dabrafenib, cisplatin, and bortezomib) [59].

The existing models for the preclinical MM research are illustrated in Table 4.

## 4. Innovative Models for Future Rare Melanoma Research

### 4.1. Modeling Biological Heterogeneity

Melanoma is known to have the highest mutation frequency among human cancers, leading to an increase in heterogeneity as the disease progresses. Heterogeneity, which is a recognized barrier in finding the best treatment for cancer patients, can be classified into interpatient heterogeneity, intertumoral heterogeneity, and intratumoral heterogeneity. Interpatient heterogeneity describes the differences in tumors between patients, while intertumoral heterogeneity refers to differences between lesions in the same patient. Intratumoral heterogeneity describes the variation in populations of cancer cells within a given tumor. Advances in molecular biology and sequencing techniques have facilitated the identification of intratumoral heterogeneity with studies revealing discrepancies in the mutation status of paired primary and metastatic lesions, including BRAF and KIT mutations. Discordant BRAF mutation status has been observed in multiple investigations, which poses a significant problem for treatment selection and response. Metastases are genetically diverse in comparison to the primary tumor or other metastases from the same patient, as demonstrated by whole-genome sequencing analysis [60,61].

Additionally, aneuploidy and whole-genome doubling occur at a higher frequency in late-stage samples compared to matched primary tumor samples. This intratumoral evolution likely enhances survival and promotes metastasis. Intratumoral heterogeneity refers to the differences between cells or subpopulations within a tumor, which can display varying phenotypic traits. Studies have found that intratumoral heterogeneity exists in all cancer types, with melanoma being the most heterogeneous. Genetic instability is one factor that contributes to intratumoral heterogeneity, with mutations leading to the presence of subpopulations with different genetic variations. Analyses such as DNA microsatellite loci analysis, sequencing, high-resolution melting analysis, immunohistochemistry, and mutation-specific PCR have been used to explore genetic heterogeneity in melanoma. Mutually exclusive mutations have been identified in different tumor cell populations within a single tumor, and some subpopulations have been found to possess survival advantages that may impact treatment outcomes. Overall, the existence of intratumoral heterogeneity highlights the importance of personalized treatment approaches that target the specific subpopulations present within a tumor [60,61]. 

The heterogeneity observed within tumors can originate from non-genetic sources such as epigenetic and phenotypic heterogeneity. Epigenetic mechanisms like DNA methylation, histone modification, and non-coding RNA regulation contribute to melanoma development. Differential methylation and histone modification were observed in clones derived from melanoma lesions, leading to varied expression of tumor-related genes and therapeutic targets. Phenotypic heterogeneity can result from the dynamic expression of certain markers in different regions in response to microenvironmental cues, disease development, treatment, or progression, without undergoing genetic alterations. The co-existence of microphthalmia-associated transcription factor (MITF) MITFhigh and MITFlow subpopulations in different regions of patient tumor samples was found, and the interaction between them was observed in a zebrafish melanoma xenograft model. The heterogeneous populations of proliferative and invasive cells were found to be more metastatic and invasive compared to single populations, suggesting cooperativity between heterogeneous populations in driving metastasis [60,61].

### 4.2. In Silico Models for Melanoma Research

In silico models refer to computer-based models that simulate biological processes or systems. In the context of melanoma, in silico models can be used to simulate the behavior of melanoma cells, predict treatment outcomes, and identify potential drug targets [62].

There are several types of in silico models of melanoma, including:

1. Genomic models: These models use genomic data to predict the behavior of melanoma cells. For example, machine learning algorithms can be trained on genomic data to predict the likelihood of melanoma progression.
2. Mathematical models: These models use mathematical equations to describe the behavior of melanoma cells. For example, mathematical models can be used to simulate the growth and metastasis of melanoma cells.
3. Multiscale models: These models integrate multiple scales of biological organization, from molecular to cellular to tissue level, to simulate the behavior of melanoma cells. For example, multiscale models can be used to simulate the interaction between melanoma cells and the immune system.

In silico platforms have evolved to simplify data analysis and have been used for melanoma genetic characterization, which has led to the discovery of predictive biomarkers and improvements in melanoma therapy. Various in silico platforms, such as The Cancer Genome Atlas, Melanoma Gene Database, and cBioPortal, are available for melanoma metastasis research. These tools offer a wide range of analyses, including comparative analysis, visualization and integrative analysis, and subclassification analysis. Intuitive platforms, such as the Single-Cell Portal, have also been created to facilitate the analysis of multi-omics data. In silico platforms offer the advantage of analyzing limited cells and samples, including micro-metastases and dormant cells, which are challenging to achieve in the clinical setting. However, comparative analysis between primary and metastatic samples is still challenging due to the lack of standardization among different institutions. Therefore, several points must be evaluated before choosing an online tool and study, and in silico platforms still require functional validation of the findings in vitro or in vivo models [63,64].

## 5. Precision Medicine for Rare Melanoma Treatment

Based on tumor stage, genetic burden, and localization, the existing melanoma therapy covers a variety of options including surgery, chemotherapy, immunotherapy, and radiotherapy [65]. However, the reduced treatment efficacy resulting from tumor resistance to conventional treatments, and the severe adverse effects that are usually related to the lack of treatment specificity [65], constitute major obstacles hindering therapy success. Moreover, significant differences in genetic drivers might occur between tumors, and individuals as well [66]. In particular, melanoma comprises a vast landscape of subtypes (e.g., UM, ALM, MM) which, besides their rarity compared to CM, possess not only particular molecular features, distinct aggressiveness levels [67], and a reduced number of experimental models that can be used for treatment development (Table 1), but also a worse survival rate among patients as a consequence of reduced response to the approved therapies [65]. Available treatment options for these cancers were comprehensively described in previous papers [68,69,70]. 

The concept of precision medicine (PM) refers to the development of individualized treatments considering several factors such as genetic, phenotypic, and environmental characteristics. In oncology, the main goal of PM is to apply specific treatment modalities that are tailored exactly to the patient’s tumor characteristics, advancing from the “one-size-fits-all” cancer therapy to a more personalized approach [66]. Further PM progress demands the consideration of the functional context of the tumor genome in every patient; therefore, an important step is the design of genomic profiles to identify specific oncogenic mutations driving cancer development [66,71]. In this part of the review, we presented next-generation sequencing (NGS) and CRISPR/Cas9 as personalized approaches for future UM, ALM, and MM management that might provide better and more durable therapeutic results for these high-risk rare melanoma subtypes [66].

NGS is a foundational method for precision oncology, providing a large amount of genomic information for a variety of applications, including cancer diagnosis, prognosis, and treatment selection [72,73]. The development of NGS technology provided a deeper understanding of the underlying molecular mechanisms of carcinogenesis through the identification of novel cancer mutations, thus offering a molecular rationale for choosing the proper targeted therapy [74]. The progress made regarding the molecular pathogenesis of melanoma has advanced the development of efficient treatments—e.g., BRAF, MEK, and KIT inhibitors—that specifically target genetic drivers [75]. Recent papers have reported the design of melanoma-specific NGS panels that describe the mutational profile of primary melanoma samples [75], or identify targetable mutations linked to treatment resistance [76]. Regarding the application of NGS in the research of rare melanoma subtypes, Afshar et al. used NGS to detect genetic mutations that predict tumor metastasis in UM [77], while Cosgarea et al. applied NGS to assess the frequency of NF1, RAS, BRAF and KIT alterations in MM samples [78]. 

CRISPR/Cas9 represents an innovative technology used to perform genome modifications to investigate gene functions in various cellular events [79]. The emergence of CRISPR/Cas9 revolutionized oncological research by enabling the study of tumor etiology, occurrence, development, and metastasis [66]. Also, by enabling the discovery of cancer targets, this method facilitates the development of efficient treatments [79]. For instance, by resorting to CRISPR/Cas9 technology, Ercolano et al. identified prostaglandin-endoperoxide synthase 2 (PTGS2) as a potential target for melanoma treatment, demonstrating that its knockdown leads to inhibited cell proliferation and migration, altered differentiation of the myeloid-derived suppressor cells, and repressed tumor development and invasion in C57BL/6 mice [79]. To develop novel treatments for UM, CRISPR/Cas9 was recently exploited to discover molecular targets whose inhibition could enhance the antitumor effect of everolimus in a synergistic manner [80]. Another relevant application of genome editing in cancer research is the development of innovative experimental models. A representative example would be the recent study performed by Hodis et al. who designed nine genetically distinct models for human melanoma, by sequentially introducing cancer-associated mutations into human melanocytes [81]. 

Ultimately, the data obtained from NGS profiles could also constitute the basis for further CRISPR/Cas9 editing of the mutated genes [66], the association of these two methods thus improving the personalized treatment of various cancers, including the uncommon melanoma subtypes (UM, ALM, and MM). 

## 6. Conclusions

In comparison to CM, the rare melanoma subtypes—UM, ALM, and MM—that are explored in this review face a scarcity of preclinical models that can be utilized for comprehending disease patterns and formulating effective treatments. Nevertheless, this review highlights the latest accomplishments in this domain, which encompasses three primary categories of experimental models: in vitro, in vivo, and in ovo. These experimental models demand increased attention in future research, such as the intention to devise innovative and highly sophisticated models that accurately mimic the characteristics of these uncommon melanoma subtypes.

Despite the advancements in the field, it is important to recognize that no single model can replicate the intricate features of these melanomas. However, by augmenting the existing library of models and amalgamating data acquired through various methodologies, including in vitro, in vivo, in ovo, and computational approaches, significant progress can be achieved in this critical area of research.

By broadening the range of available models and bolstering their accuracy, researchers can gain a deeper understanding of these rare melanoma subtypes and their unique biological and pathological characteristics. Combined with the development of innovative modalities (NGS and CRISPR/Cas9) that provide personalized approaches in cancer treatment, this improved understanding will ultimately facilitate the development of more targeted and effective therapeutic strategies, paving the way for better patient outcomes and a more comprehensive understanding of these lesser-known melanoma subtypes. Consequently, the continuous refinement and expansion of the existing model library, as well as the constant investigation of advanced and precise therapy options, are crucial in driving advancements in this specialized field of research.

## Figures and Tables

**Table 1:** Comparative overview of the characteristics of CM and rare melanoma types (UM, ALM, MM).

Melanoma Type	Localization	Incidence (% of Total Melanoma Cases)	Genetic Drivers	Available Experimental Models	References
CM	Skin	90%	BRAF, NF1, NRAS, TERT and CDKN2A mutations	Cell lines, spheroids, organoids, melanoma-on-a-chip, reconstructed tissue, GEMs, STMs, ZMMs, PDXs, Chick embryo CAM, etc.	[23,24,32,33]
UM	Iris, choroid, ciliary body	5%	BAP1, GNAQ, GNA11, EIF1AX, SF3B1 mutations	Cell lines, Co-cultures, spheroids, ZMMs, PDXs, Chick embryo CAM.	[34,35,36,37,38]
ALM	Palms, soles, or nail beds	2–3%	KIT mutations	Cell lines, PDXs, ZMMs	[7,19,39,40]
MM	The nasal cavity, the mucous covering the mouth, vagina, anus, gastrointestinal tract, urinary tract, biliary bladder	0.8–3.7%	KIT, GNAQ/11 mutations	Cell lines, Spheroids, PDXs	[8,21,22]

CM = cutaneous melanoma; UM = uveal melanoma; ALM = acral lentiginous melanoma; MM = mucosal melanoma; GEMs = genetically engineered models; STMs = syngeneic transplantation models; ZMMs = zebrafish melanoma models; PDXs = patient-derived xenografts.

**Table 2:** Summary of the identified preclinical models used in UM research and their applications.

Experimental Models for UM	Examples	Applications	References
Cell Lines	Primary (92.1, MP41, MP46, MEL270, OCM-1, OCM-3, OCM-8, SP6.5) and metastatic (OMM1, OMM2.3, OMM2.5, MUM2C) cell lines	Anti-cancer drug screening; Genetic characterization for further preclinical studies	[18,43,44,45]
Co-culture	92.1 UM cells + human retinal pericytes (HRPC)	Evaluation of the interaction between UM cells and a TME cellular component	[35]
	Mel270 and OMM2.3 UM cells + LX-2 hepatic stellate cells	Investigation of the role of hepatic microenvironment on UM growth and survival	[47]
Spheroids	Spheroids derived from 92.1, Mel270, UPMD2, UPMD3, MP46, MM28, OMM1 cell lines	Evaluation of electrochemotherapy with bleomycin as treatment for UM	[36,37]
PDX Mouse Model	UM xenografts in SCID mice	Assessment of the pharmacological effects of anti-cancer drugs (fotemustine, and dacarbazine/temozolomide)	[51]
	Orthotopic PDX in NSG mice using liver metastatic metastases	Preclinical research studies of UM	[52]
Zebrafish	Orthotopic 92.1 xenograft in zebrafish embryos	Screening of anti-cancer drugs	[50]
	Mel270, OMM2.3, OMM2.5, OMM1, 92.1 xenografts in zebrafish embryos	Evaluation of UM cell behavior (i.e., interaction with host environment, proliferation, migration, and metastasis)	[49]
Chick Embryo CAM	92.1 cells—Matrigel grafts	Evaluation of tumor growth; Assessment of ECT as a potential treatment for UM	[36]

UM = uveal melanoma; SCID = severe combined immunodeficiency disease; NSG = NOD/SCID/IL-2Rγnull; CAM = chorioallantoic membrane.

**Table 3:** Summary of the identified preclinical models used in ALM research and their applications.

Experimental Models for ALM	Examples	Applications	References
Cell Lines	Primary (WM3211, MMG1, SMYM), and metastatic (SM2-1, Mel18) cell lines	Genomic characterization for further preclinical studies	[39]
	XYAM-1, XYAM-2, XYAM-3, and XYAM-4	Investigation of the mutational profile for further preclinical studies	[40]
PDX Mouse Model	XYAM-4 xenograft in NSG mice	Evaluation of the proliferative and metastatic potential of ALM cells in vivo	[40]
Zebrafish	Transgenic zebrafish model	-	[54]

ALM = acral lentiginous melanoma; PDX = patient-derived xenograft; NSG = NOD/SCID/IL-2Rγnull.

**Table 4:** Summary of the identified preclinical models used in MM research and their applications.

Experimental Models for MM	Examples	Applications	References
Cell Lines	MM9H-1 cell line	High-throughput drug screening of anti-cancer agents	[59]
	COMM-1 and COMM-2 cell lines	Investigation of the possible association between melanoma heterogeneity and metastasis	[58]
Spheroids	Spheroids derived from the MM9H-1 cell line	-	[59]
PDX Mouse Model	MM9H-1 xenograft in BALB/c nude mice (subcutaneous and orthotopic inoculations)	Evaluation of tumor metastasis and tumorigenesis in vivo	[59]

MM = mucosal melanoma; PDX = patient-derived xenograft.

## Data Availability

Not applicable.
